# Long noncoding RNA LINC00460 conduces to tumor growth and metastasis of hepatocellular carcinoma through miR-342-3p-dependent AGR2 up-regulation

**DOI:** 10.18632/aging.103278

**Published:** 2020-06-03

**Authors:** Han Hong, Chengjun Sui, Tao Qian, Xiaoyong Xu, Xiang Zhu, Qiang Fei, Jiamei Yang, Minhui Xu

**Affiliations:** 1 Department of Hepato-Pancreato-Biliary Surgery, The Affiliated Suzhou Hospital of Nanjing Medical University, Suzhou 215001, China; 2 Department of Special Treatment I and Liver Transplantation, Shanghai Eastern Hepatobiliary Surgery Hospital, Shanghai 200438, China; 3 Department of General Surgery, Affiliated Hospital of Integrated Traditional Chinese and Western Medicine, Nanjing University of Chinese Medicine, Nanjing 210028, China

**Keywords:** hepatocellular carcinoma, LINC00460, miR-342-3p, AGR2, tumor growth

## Abstract

Hepatocellular carcinoma (HCC) is the fifth most common malignant tumor in the world. It ranks third among cancer-induced deaths worldwide and has the characteristics of high metastasis and high recurrence rate. Long non-coding RNA (LncRNA) LINC00460 is significantly up-regulated in multiple types of cancers and is closely related to the progression of tumors. However, effects of LINC00460 and corresponding regulatory path in HCC are still poorly investigated.

In our study, we found that expression of LINC00460 was up-regulated in HCC tissues and cell lines compared with the control. Then we revealed that knockdown of LINC00460 suppressed cell proliferation and cell mobility and induced cell apoptosis in HCC cells. Further study demonstrated that knockdown of LINC00460 suppressed the progression of HCC by elevating the expression of microRNA (miRNA, miR)-342-3p. Besides that, metastasis marker, Anterior gradient homolog 2 (AGR2) was found to be a target of miR-342-3p and overexpression of AGR2 promoted the progression of HCC. Finally, the *in vivo* experiments further verified the anti-tumor effects of LINC00460 / miR-342-3p / AGR2 axis in HCC.

The LINC00460 / miR-342-3p / AGR2 axis exerts anti-tumor effect in HCC *in vitro* and *in vivo*, consolidating and expanding the research about targeted gene therapy for early diagnosis and treatment of HCC.

## INTRODUCTION

Hepatocellular carcinoma (HCC) is one of the malignant tumors that seriously threaten human life. It is the fifth most common malignant tumor and ranks third among the causes of cancer deaths worldwide [1, 2]. There are about 1 million people expected to die from HCC yearly. Due to the lack of accurate and timely early diagnosis to differentiate HCC from cirrhosis, a sharp increase in HCC patients was induced [3]. Metastasis and recurrence of HCC are the main causes of death and also are the greatest challenge for clinical treatment and prevention of HCC [4]. Therefore, searching for biomarkers for early diagnosis, the prediction of metastasis and targeted therapy of HCC has great theoretical and clinical significance.

Long non-coding RNA (lncRNA) is a class of non-coding RNA molecules with length greater than 200 bp. LncRNA acts as an important regulator of chromatin modification, transcription and post-transcriptional regulation through interacting with DNA, RNA and protein molecules [5]. At present, many lncRNAs are proved to be imbalanced in HCC and are associated with the progression of HCC. For example, Yu WW et al. reported that lncRNA ITGB1 was up-regulated in HCC and it promoted the metastasis and epithelial to mesenchymal transition (EMT) via regulating ZEB1 [6]. Long non-coding RNA LINC00460 locates at chromosome 13q33.2 and is significantly up-regulated in multiple types of cancers [7]. Li K et al. reported that lncRNA LINC00460 was significantly up-regulated in NSCLC tumors and promoted cell migration and invasion through inducing EMT in lung cancer cells [8]. However, effects of LINC00460 in HCC have not been widely explored before.

MicroRNAs (miRNAs) are short sequence of nucleotides with only 20-24 nt, which are widely expressed in multicellular eukaryotic organisms. MiRNAs regulate its target genes by binding to the 3' un-translated region of target messenger RNAs (mRNAs) [9, 10]. A large number of studies have found that miRNA can regulate many biological processes, such as cell proliferation, differentiation and apoptosis, thus participating in the regulation of various cancers. Liu W et al. reported that miR-342-3p acted as a tumor suppressor in liver cancer through inhibition of IGF-1R-mediated Warburg effect [11]. Besides that, Wang F et al. revealed that overexpression of LINC00460 promoted cell proliferation, migration and invasion in gastric cancer by targeting miR-342-3p [12]. In our present study, we explored effects of miR-342-3p in HCC cells and tried to find relationship between miR-342-3p and LINC00460.

Anterior gradient homolog 2 (AGR2) locates at chromosome 7p21.3 and is a secretory protein existing in human breast, colon, lung, pancreas and other glands [13]. Previous studies have pointed out that overexpression of AGR2 is closely associated with the growth, metastasis and survival of tumors [14]. Yu H et al. reported that overexpression of AGR2 increased the invasion ability of HCC cells *in vitro* and *in vivo* [15]. However, potential interactions between LINC00460 / miR-342-3p / AGR2 during the progression of HCC remain indistinct. To make up for the research, our present study aimed to explore the underlying regulatory mechanism of LINC00460 / miR-342-3p / AGR2 in the proliferation, apoptosis and mobility of HCC *in vivo* and *in vitro*, providing research basis for novel therapeutic targets in early diagnosis and treatment of HCC.

## RESULTS

### Up-regulated LINC00460 is found in human HCC tissues and cell lines

**Figure 1:**
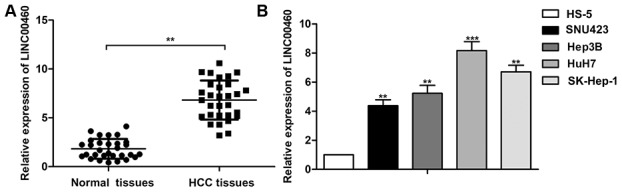
**Up-regulated LINC00460 is found in human HCC tissues and cell lines.** (**A**) Relative expression of LINC00460 in HCC tissues and the normal tissues was detected through qRT-PCR. (**B**) Relative expression of LINC00460 in HCC cells lines (SNU423, Hep3B, HuH7 and SK-Hep-1) and the control HS-5 cells was detected through qRT-PCR. ^**^P < 0.01, ^***^P < 0.001 vs the control group.

As shown in Figure 1A, relative expression of LINC00460 was found up-regulated in HCC tissues compared with the normal tissues (about 3-folds). Besides that, relative expression of LINC00460 in HCC cells lines (SNU423, Hep3B, HuH7 and SK-Hep-1) was also obviously higher than that in the control HS-5 cells (about 4-8 folds, Figure 1B). Thus, obviously up-regulated expression of LINC00460 may be related to the progression of HCC.

### Knockdown of LINC00460 suppresses HCC cell proliferation, migration and invasion and promotes cell apoptosis

**Figure 2:**
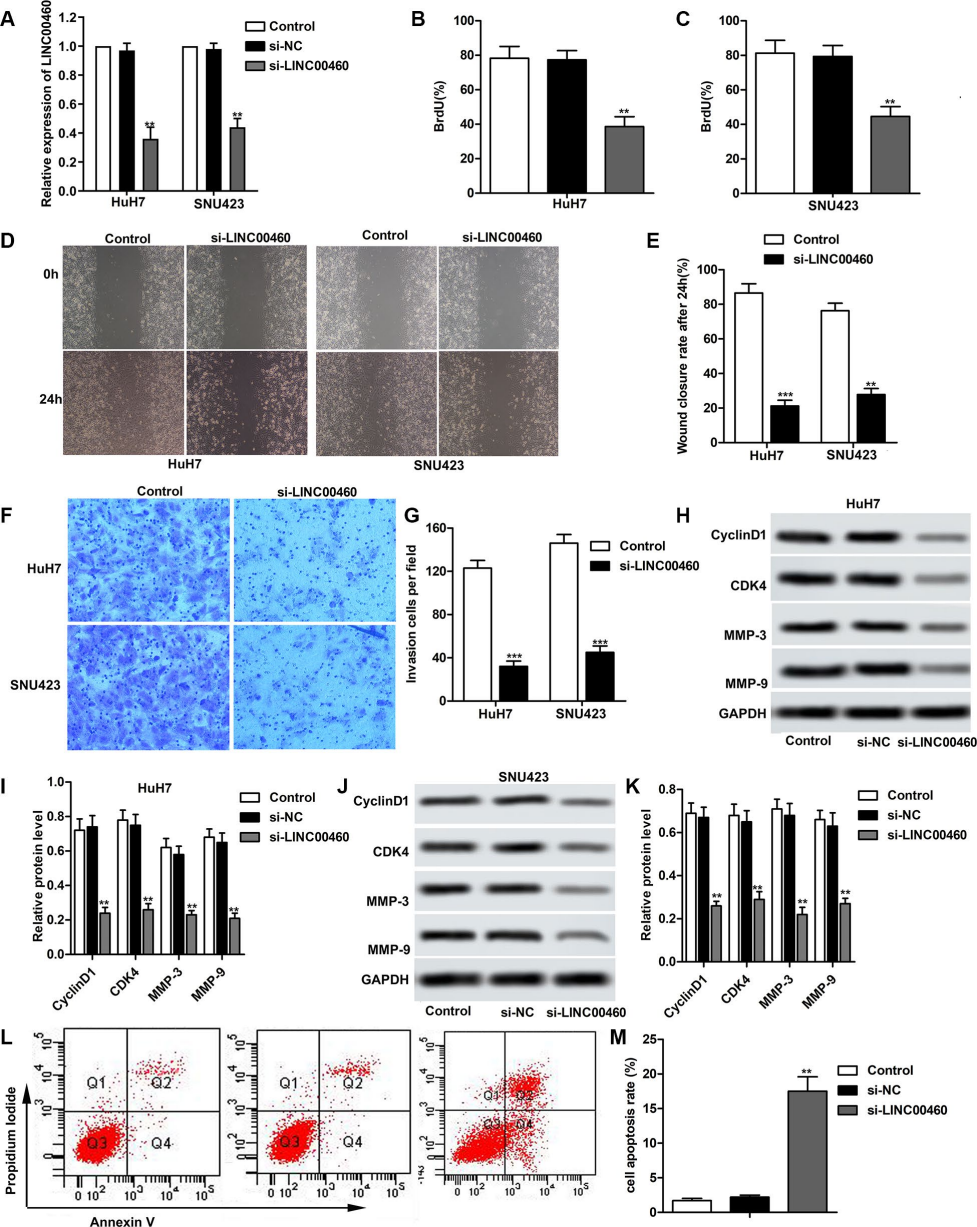
**Knockdown of LINC00460 suppresses HCC cell proliferation, migration and invasion.** Specific si-LINC00460 was transfected into HuH7 and SNU423 cells, respectively. si-NC was used as a blank. (**A**) Relative expression of LINC00460 in HuH7 and SNU423 cells was detected through qRT-PCR. (**B**, **C**) Bromodeoxyuridine assay was used to value cell proliferation in HuH7 and SNU423 cells, respectively. (**D**, **E**) Wound healing assay was conducted to value cell migration ability. Data statistics was also shown. (**F**, **G**) Transwell invasion assay showed the invasive cells in each group. Data statistics was also shown. (**H**, **I**) Western blot showed the expression of cell proliferation related proteins CyclinD1 and CDK4 and cell migration related proteins MMP-3 and MMP-9 in HuH7 cells. Data statistics was also shown. (**J**, **K**) Western blot showed the expression of CyclinD1, CDK4, MMP-3 and MMP-9 in SNU423 cells. Data statistics was also shown. (**L**) Flow cytometry was conducted to value cell apoptosis. (**M**) Cell apoptosis rate was calculated and presented. ^**^P < 0.01, ^***^P < 0.001 vs the control group.

In order to investigate the effects of LINC00460 in HCC progression, specific si-LINC00460 was transfected into HuH7 and SNU423 cells respectively as shown in Figure 2A. At the same time, si-NC was used as a blank. Then, Bromodeoxyuridine assay was used to value cell proliferation as shown in Figure 2B, 2C and we found that cell proliferation was both suppressed in si-LINC00460 group in HuH7 and SNU423 cells compared with the control. The results of the wound healing assay and the data statistics in Figure 2D, 2E showed that knockdown of LINC00460 effectively suppressed cell migration. Besides that, the transwell invasion assay showed less invasive cells in the si-LINC00460 group than the control group (Figure 2F–2G). Results of western blot also showed that knockdown of LINC00460 suppressed the expression of cell proliferation related proteins CyclinD1 and CDK4, inducing obvious cell cycle arrest. The expression of cell migration related proteins MMP-3 and MMP-9 was also greatly suppressed by the transfection of si-LINC00460 in both HuH7 and SNU423 cells (Figure 2H–2K). And beyond this, cell apoptosis was valued through flow cytometry and it showed that silencing of LINC00460 promoted cell apoptosis comparing to the control (Figure 2L–2M). Thus, knockdown of LINC00460 effectively suppresses HCC cell proliferation, migration and invasion and promotes cell apoptosis.

### LINC00460 interacts with miR-342-3p in HCC cells

**Figure 3:**
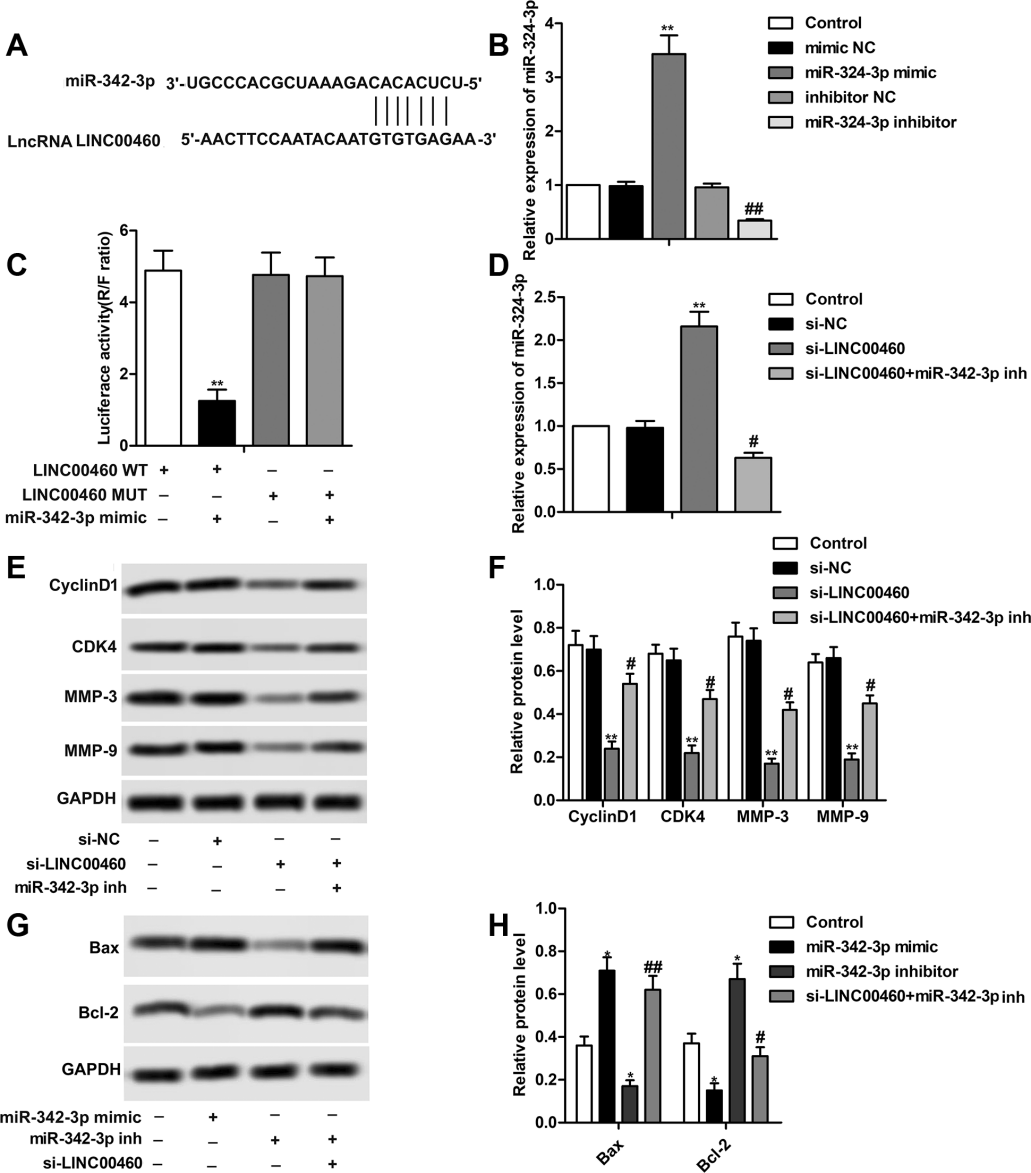
**LINC00460 interacts with miR-342-3p in HCC cells.** (**A**) Bioinformatics prediction showed the complementary sequence between LINC00460 and miR-342-3p. (**B**) Relative expression of miR-342-3p in HuH7 cells was detected through qRT-PCR. (**C**) Luciferase reporter assay was conducted to verify the targeting relationship between LINC00460 and miR-342-3p. (**D**) Relative expression of miR-342-3p in HuH7 cells was detected through qRT-PCR. (**E**, **F**) Western blot showed the expression of CyclinD1, CDK4, MMP-3 and MMP-9 in SNU423 cells. Data statistics was also shown. (**G**, **H**) Western blot showed the expression of Bax and Bcl-2 in SNU423 cells. Data statistics was also shown. ^*^P < 0.05, ^**^P < 0.01vs the control group. ^#^*P* < 0.05, ^##^*P* < 0.01 vs the si- LINC00460 group.

The result of bioinformatics prediction showed that there existed complementary sequence between LINC00460 and miR-342-3p (Figure 3A). Specific miR-342-3p mimic and miR-342-3p inhibitor were designed and synthesized to up-regulate or down-regulate the expression level of miR-342-3p by transfecting into HuH7 cells as shown in Figure 3B. The results of the luciferase reporter assay showed that the combination of miR-342-3p mimic with LINC00460 WT largely decreased luciferase activity compared with the other groups, further indicating that there exists targeting relationship between miR-342-3p mimic with LINC00460 (Figure 3C). The results in Figure 3D showed that relative expression of miR-342-3p was elevated in the si-LINC00460 group but was then suppressed with the combination of miR-342-3p inhibitor. Then we found that the inhibiting effect of si-LINC00460 on CyclinD1, CDK4, MMP-3 and MMP-9 was largely weakened by the adding of miR-342-3p inhibitor (Figure 3E, 3F). In addition, we found that cell apoptosis was largely enhanced by miR-342-3p mimic and was strongly inhibited by miR-342-3p inhibitor. At the same time, suppressed cell apoptosis by miR-342-3p inhibitor was then elevated in the existence of si-LINC00460 (Figure 3G, 3H). The above results showed that si-LINC00460 suppressed cell proliferation and cell mobility and promoted cell apoptosis by elevating the expression of miR-342-3p.

### Knockdown of LINC00460 suppresses AGR2 expression by targeting miR-342-3p

**Figure 4:**
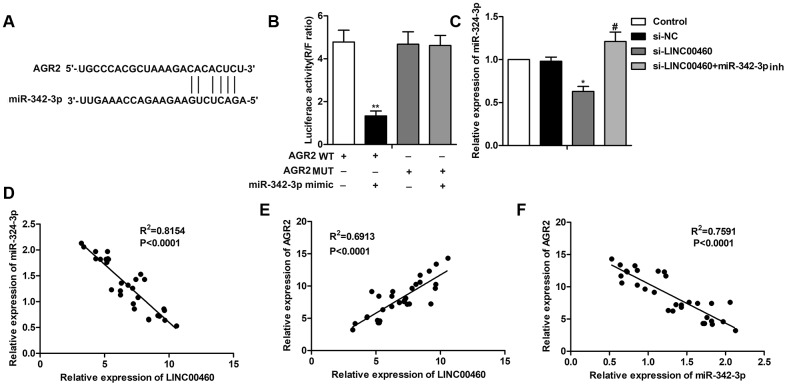
**Knockdown of LINC00460 suppresses AGR2 expression by targeting miR-342-3p.** (**A**) Bioinformatics prediction showed the complementary sequence between AGR2 and miR-342-3p. (**B**) The targeting relationship between AGR2 and miR-342-3p was further verified by the luciferase reporter assay. (**C**) Relative expression of AGR2 was detected through qRT-PCR. (**D**–**F**) The trend lines between the level of LINC00460, AGR2 and miR-342-3p. ^*^P < 0.05, ^**^P < 0.01vs the control group. ^#^*P* < 0.05 vs the si- LINC00460 group.

Further study showed that there also exist complementary sequences between AGR2 and miR-342-3p (Figure 4A). The targeting relationship between AGR2 and miR-342-3p was further verified by the luciferase reporter assay which showed that the combination of AGR WT and miR-342-3p mimic largely decreased luciferase activity compared with the control group (Figure 4B). The results in Figure 4C showed that the level of AGR2 was suppressed by si-LINC00460 and was then elevated with the combination of miR-342-3p inhibitor. The trend lines in Figure 4D–4F also showed that the level of LINC00460 was positively related to AGR2 and negatively related to the expression of miR-342-3p. In addition, the level of AGR2 was negatively related to the expression of miR-342-3p. Thus, the above results showed that knockdown of LINC00460 suppressed AGR2 expression by targeting miR-342-3p.

### Overexpression of AGR2 promotes the progression of HCC

**Figure 5:**
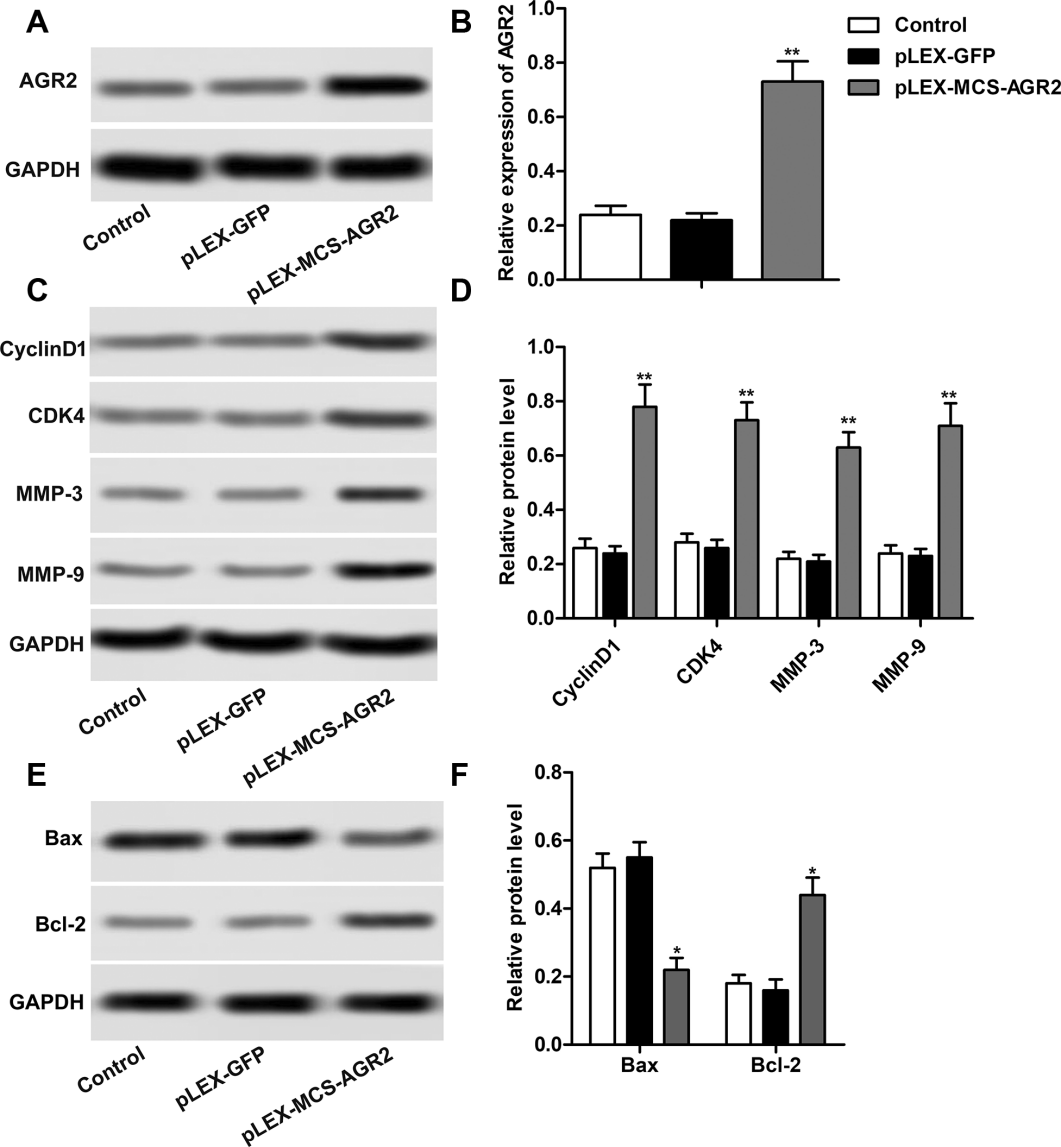
**Overexpression of AGR2 promotes the progression of HCC.** (**A**, **B**) Western blot showed the expression of AGR2 and data statistics was also shown. (**C**, **D**) Western blot showed that expression of CyclinD1, CDK4, MMP-3 and MMP-9. Data statistics was also shown. (**E**, **F**) Western blot showed that expression of Bax and Bcl-2. Data statistics was also shown. ^*^P < 0.05, ^**^P < 0.01vs the control group.

In order to investigate the effect of AGR2 in the progression of HCC, recombinant plasmid pLEX-MCS-AGR2 was constructed and transfected into HuH7 cells to up-regulate the expression of AGR2 as shown in Figure 5A, 5B. Then the results of western blot showed that overexpression of AGR2 induced elevation of CyclinD1, CDK4, MMP-3 and MMP-9, indicating that overexpression of AGR2 promoted the proliferation and cell mobility of HCC (Figure 5C, 5D). Detection about cell apoptosis also showed that overexpressed AGR2 markedly elevated expression of Bax and suppressed expression of Bcl-2 (Figure 5E, 5F). The above results showed that overexpression of AGR2 promoted the progression of HCC.

### Knockdown of LINC00460 suppressed HCC progression *in vivo*

**Figure 6:**
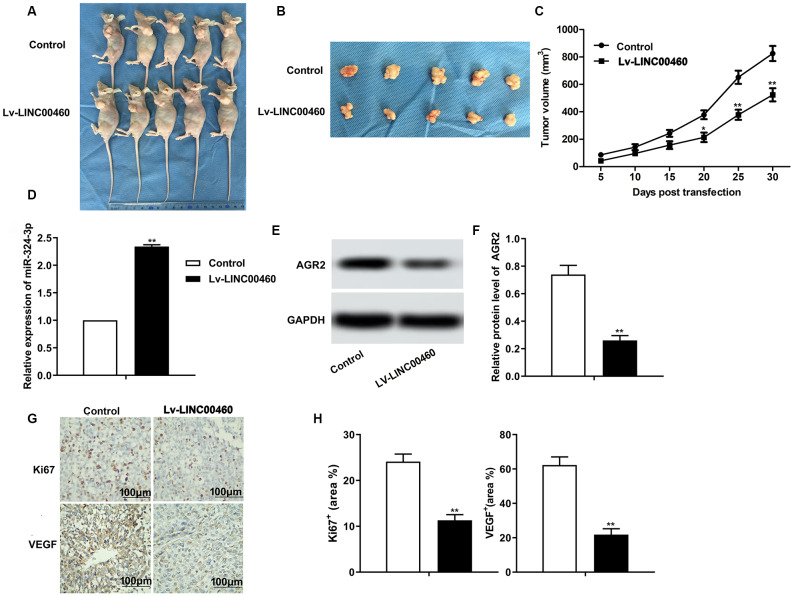
**Knockdown of LINC00460 suppresses HCC progression *in vivo*.** (**A**, **B**) Pictures about tumor growth in the Lv-LINC00460 group and the control group was shown (**C**). Corresponding tumor growth curve. (**D**) The level of miR-342-3p was detected through qRT-PCR. (**E**, **F**) The expression of AGR2 was detected through western blot and data statistics was also shown. (**G**–**H**) Immunohistochemical (IHC) was conducted to examine the expression of Ki67 and VEGF in tissues. ^*^P < 0.05, ^**^P < 0.01vs the control group.

The *in vivo* experiments were further conducted to explore the effects of LINC00460 on HCC tumor progression. The result in Figure 6A–6C showed that knockdown of LINC00460 by transfected with Lv-LINCC460 suppressed the growth of HCC tumor compared with the control. The level of miR-342-3p was also elevated by Lv-LINCC460 as shown in Figure 6D. In addition, the expression of AGR2 was also decreased by Lv-LINCC460 (Figure 6E–6F). The results of immunohistochemical (IHC) also showed that knockdown of LINC00460 suppressed the expression of Ki67 and VEGF, thus inhibiting the progression of HCC *in vivo* (Figure 6G–6H). Thus, we concluded that knockdown of LINC00460 suppressed the progression of HCC *in vivo* by inhibiting cell proliferation and cell mobility.

## DISCUSSION

Hepatocellular carcinoma (HCC) is currently recognized as a refractory malignant tumor with the characteristics of rapid progress, easy metastasis and recurrence and short survival time, etc. Because of the occult incidence of HCC, most patients are found in advanced stage, thus losing the best chance of operation or liver transplantation. Most of the present treatments of HCC such as hepatectomy, liver transplantation, transarterial chemoembolization radiotherapy have many adverse reactions and result in low overall survival rate [16]. Molecular targeted therapy in cancer has achieved much progress now, so it is urgent to find accurate and effective therapeutic targets for HCC treatment.

Previous study has revealed the close relationship between abnormal expression of lncRNA and human health, thus lncRNA is gaining more and more attention in cancer research. Researchers pointed out that expression of lncRNA was significantly correlated with cancer metastasis and the overall survival rate of cancer patients [17]. LncRNA LINC00460 is found up-regulated in kinds of cancers and is closely associated with cancer progression. For example, Liang Y et al. reported that LINC00460 worked as an oncogene in esophageal squamous cell carcinoma and overexpression of LINC00460 was positively correlated with TNM stage, lymph node metastasis, and predicted poor prognosis of esophageal squamous cell carcinoma [18]. Ye JJ et al. also demonstrated that LINC00460 was increased in lung adenocarcinoma and overexpression of LINC00460 contributed to lung adenocarcinoma progression by regulating the miR-302c-5p/FOXA1 signal pathway [19]. However, the effects of LINC00460 in HCC have not been explored yet. In our study, we found that the expression of LINC00460 was increased in HCC tissues and cell lines. The knockdown of LINC00460 effectively suppressed cell proliferation, migration and invasion and induced cell apoptosis. Thus, we concluded that LINC00460 may act as a novel biomarker for HCC diagnosis and demonstrated that silenced LINC00460 had anti-tumor effect in HCC.

MiRNAs not only participate in the normal growth, metabolism and development of organisms, but also are associated with many biological processes [9]. Previous studies summarized that there exist several different regulation mode between lncRNAs and miRNAs. LncRNA competes with 3'-UTR of target mRNA or miRNAs or act as a competitive endogenous RNA (ceRNA) to inhibit the expressions of miRNAs [20, 21]. LncRNAs can also act as potential pri-microRNAs to produce mature miRNAs, indirectly regulating the expressions of target genes [22]. In addition, lncRNA can indirectly regulate the expression of miRNAs through other target proteins. The interaction between miRNAs and lncRNAs is not only related with the occurrence and progression of cancer, but also acts as important targets in the process of cancer research. Wang SH et al. reported that overexpressed lncRNA H19 acted as ceRNA to elevate the expression of FOXM1 by competitively binding to miR-342-3p in gallbladder cancer [23]. In our study, we revealed the targeting relationship between LINC00460 and miR-342-3p and pointed out that the knockdown of LINC00460 suppressed cell proliferation and mobility and induced cell apoptosis by elevating miR-342-3p.

AGR2 is found elevated in numerous types of cancer and it has been reported as a novel biomarker with a potential oncogenic role. For example, Zhang J et al. reported that AGR2 expression was significantly elevated in gastric cancer and high level AGR2 was associated with the location and size of gastric cancer, depth of invasion, TNM stage, lymphatic metastasis, vessel invasion and distant metastasis [24]. Besides that, Alavi M et al. reported that high expression of AGR2 in lung cancer was prediction of poor survival [25]. To explain associated mechanism, Yu H et al. explained that AGR2 may be a pro-metastatic protein in HCC through proteomic study [15]. Moreover, some other researchers pointed out that overexpressed AGR2 may exert carcinogenesis through inhibiting p38 MAPK and preventing p53 activation by phosphorylation [26]. Thus, the activity and related regulation of AGR2 is worthy of further study for acting as worthwhile therapeutic target in cancers including HCC. Consistent with previous research, we found that, via AGR2, up-regulation of LINC00460 expression and down-regulation of miR-342-3p expression could facilitate the proliferation and mobility of HCC cells.

The effects of lncRNA LINC00460 *in vivo* have also been explored in kinds of cancers before. For example, Zhang S et al. reported that down-regulation of lncRNA LINC00460 expression suppressed tumor growth *in vivo* in gastric cancer [27]. Overexpressed AGR2 was also demonstrated to contribute to growth and angiogenesis of glioblastoma in vitro and in tumor xenografts [28]. In our study, we found that growth and metastasis of tumor xenograft was obviously suppressed by silenced LINCC00460. Besides that, the level of miR-342-3p was elevated and the expression of AGR2 was suppressed by Lv-LINCC00460 just as in cellular level. Thus, knockdown of LINCC460 successfully suppressed the progression of HCC *in vivo*.

Taken together, silenced LINC00460 suppressed cell proliferation, invasion, and metastasis and induced cell apoptosis in HCC by inhibiting the expression of miR-342-3p and up-regulating the expression of AGR2. Our present study for the first time comprehensively explored effects of the LINC00460/miR-342-3p/AGR2 axis on call proliferation, apoptosis, invasion and migration in HCC in vitro and in vivo, providing theoretical basis for genetic therapy of HCC. However, further studies such as related signal pathway, are also needed to explore in HCC. Thus, we would conduct more scrupulously and all-round experiments in our follow-up study, in order to provide more scientific research findings with clinical application value.

## MATERIALS AND METHODS

### Tissue samples collection

HCC tissues and normal control tissues were obtained from 60 patients who underwent surgical treatment between May 2016 and February 2018 at the Affiliated Suzhou Hospital of Nanjing Medical University. Tissue samples were snap frozen in liquid nitrogen immediately after surgical resection and stored at -80°C until use. All patients related to our present study gave written informed consents. This study was approved by the Medical Ethics Committee of the Affiliated Suzhou Hospital of Nanjing Medical University.

### Cell culture

The control cell, HS-5, a non-cancer bone marrow stromal cell line, and four HCC cell lines (SNU423, Hep3B, HuH7 and SK-Hep-1) were purchased from the Chinese Academy of Sciences Cell Bank (Shanghai, China). All cells were cultured in RPMI-1640 (Thermo Fisher Scientific, Waltham, MA, USA) containing 10 % fetal bovine serum (FBS, HyClone, Invitrogen, Camarillo, CA, USA), and were maintained in a humidified incubator at 37 °C in the presence of 5 % CO2.

**Quantitative real-time polymerase chain reaction (qRT-PCR)**

Total RNA was extracted from tissues and cells using the Trizol reagent (Invitrogen) and the RNA was reversely transcribed into cDNAs using the Primer-Script one step RT-PCR kit (TAKARA, Dalian, China). qRT-PCR was performed using ABI 7900 RT-PCR system with a SYBR Premix Ex Taq Kit (TaKaRa). β-actin and U6 SnRNA were used as the internal control of the mRNA or miRNA, respectively. The primer sequences used were as follows: LINC00460 (forward: 5’-GCATGCACACTTCTCGGCTA-3’; reverse: 5’- GAATGCGTCTTCTTTCCCACG-3’), AGR2 (forward: 5’- ACAAAGGACTCTCGACCCAAA-3’; reverse: 5’-GTGGGCACTCATCCAAGTGA-3’), MiR-342-3p (forward: 5’-TCCTCGCTCTCACACAGAAATC-3’; reverse: 5’- TATGGTTGTTCACGACTCCTTCAC-3’; Relative gene expression was quantified by 2^−ΔΔCt^ method.

### Cell transfection

The HuH7 and SNU423 cell lines were selected for further experiments because the two have the highest and the lowest level of LINC00460 among the four hepatoma cell lines we used, which have certain representativeness. For cell transfection, HuH7 and SNU423 cells were seeded into 96-well plate until reach a 60% confluence state. Si-LINC00460, si-NC, miR-342-3p mimic, mimic NC, miR-342-3p inhibitor and the inhibitor NC (GenePharma, Shanghai, China) were transfected into HuH7 and SNU423 cells respectively as indicated using Lipofectamine 2000 reagent (Life Technologies Corporation, Carlsbad, CA, USA) according to manufacturer’s protocol.

The AGR2 overexpression vector pLEX-MCS-AGR2 was constructed and pLEX-GFP was used as a control. Recombinant plasmids pLEX-MCS-AGR2 and pLEX-GFP were transfected into cells using Lipofectamine 2000 reagent respectively. Overexpression efficiencies were valued through western blot.

Lentiviral short hairpin RNA (shRNA) LINC00460 expression vectors (Lv-LINC00460) (shRNA 1: 5’-AGACCTAATAGCCAATAAG-3’; shRNA 2: 5'-GCTAAGACCTAATAGCCAATA-3'; shRNA 3: 5’-GCCAACTTCAAGCCATTCATTGTTA-3’;) were designed and purchased from Sigma-Aldrich. Sequences of the control: 5'-GTTCTCCGAACGTGTCACGT-3'. The LINC00460 shRNA 2 which had the best interference effect was selected in our subsequent experiment. Lv-LINC00460 was also transfected into cells using Lipofectamine 2000 reagent according to manufacturer’s protocol.

### Proliferation assay

Cell proliferation was examined by using the Bromodeoxyuridine (BrdU, Sigma-Aldrich, 1 mg/mL) which was added to the cultured cells 3h before analysis. At least 1*10^3^ cells were counted in each condition and each condition contains at least five replicates.

### Wound healing assays

Briefly, transfected cells were cultured in six-well plates (5×10^4^ cells per well). At 90%–95% confluence, the monolayer of cells was scratched by a sterile plastic micropipette tip to create a wound and the destroyed cells were washed away with sterile PBS gently. Then the cells were cultured under standard conditions for another 24 hours. Then, cell migration was observed and the images were captured at 0 h and 24 h with an inverted microscope. All experiments were carried out in triplicate.

### Transwell invasion assays

The cell invasive assay was performed in the Transwell chamber (8 *μ*m pore size; BD Biosciences, Franklin Lakes, NJ). Approximately 1 × 10^5^ transfected HuH7 and SNU423 cells in 200 *μ*L serum-free medium were seeded in the upper chamber containing Matrigel and the lower chamber was supplemented with RPMI 1640 containing 10% FBS. 48 h later, the non-invasive cells on the upper surface of the chamber were removed by a cotton-tipped swab. The invasive cells on the lower surface of the chamber were fixed in 70% ethanol for 30 minutes and stained with 0.1% crystal violet for 10 minutes. The number of invasive cells was counted in five randomly selected fields using a microscope (Olympus Corporation, Tokyo, Japan).

### Western blot

The proteins in cells and tissues were extracted and separated by SDS-PAGE. Western blot analysis was performed according to standard procedures. The membranes were incubated with the primary antibodies overnight at 4°C followed by corresponding secondary antibodies for 2 h at room temperature. Protein bands were enhanced with chemiluminescence reagents and were visualized with ECL Western Blotting Substrate (Thermo Scientific, Hudson, NH). Protein levels were normalized to *β*-actin.

### Flow cytometry

Cell apoptosis was determined by flow cytometry (BD, UA). Firstly, HuH7 and SNU423 cells were stained with an apoptosis detecting kit (Invitrogen, USA). Cell apoptosis rate was calculated based on the number of Annexin-V positive cells and were analyzed by CellQuest software (BD Bio-sciences, San Jose, CA, USA) according to the manufacturer’s protocols.

### Bioinformatic prediction

In order to evaluate potential target genes of LINC00460 and AGR2, the following online miRNA target prediction algorithms were used: miRanda (http://www.microrna.org/microrna/home.do), TargetScan (http://www.targetscan.org/vert_71/) and Microcosm Targets (http://www.ebi.ac.uk/enright-srv/microcosm/htdocs/targets/v5/).

### Luciferase reporter assay

To verify the targeting relationship between LINC00460 and miR-342-3p, HuH7 cells were seeded in a 24-well plate and co-transfected with LINC00460 WT or LINC00460 MUT and miR-342-3p mimic using Lipofectamine 2000 (Invitrogen).

To verify the targeting relationship between AGR2 and miR-342-3p, HuH7 cells were seeded in a 24-well plate and co-transfected with agr2 WT or AGR2 MUT and miR-342-3p mimic using Lipofectamine 2000 (Invitrogen).

The cells were harvested 24 h after transfection. Luciferase activities were analyzed by using the Dual-Luciferase Reporter assay kit according to the manufacturer's instructions (Promega).

### Xenograft mouse model

The animal experiments were approved by The Affiliated Suzhou Hospital of Nanjing Medical University in accordance with the NIH Guide for the Care and Use of Laboratory Animals and principles of the Declaration of Helsinki. 6 week-old female BALB/c nude mice were used for animal studies. HuH7 cells were stably transfected with Lv-LINC00460 or empty vector. 2×10^5^ HuH7 cells were subcutaneously injected into either side of flank area of each mouse. 10 mice were divided into the control group and the Lv-LINC00460 groups randomly. The tumor volumes were calculated every 5 days post injection according to the standard formula: tumor volumes (mm^3^) = length × width^2^/2. The mice were sacrificed after 30 days post-injection and tumors were collected for the following experiments.

### IHC

The Immunohistochemical detection of Ki67 and VEGF was conducted as previously described [29]. The 5 μm-thick paraffin sections were incubated with Ki67 and VEGF primary antibody respectively (Cell Signaling Technology) at 4°C overnight. After incubated with corresponding HRP-conjugated secondary antibody for 1 h at room temperature, DAB (Beyotime Institute of Biotechnology, Jiangsu, China) system was used for further detection.

### Statistical analysis

All results are presented as mean ± S.D. SPSS 18.0 and GraphPad Prism 5.0 statistical software was utilized for the analysis. The difference between two groups was analyzed using a two tailed Student’s t-test. Analysis of a variance was used to analyze differences among more than 2 groups. *P* value < 0.05 was considered as statistically significant.
